# Imbalances in the Hsp90 Chaperone Machinery: Implications for Tauopathies

**DOI:** 10.3389/fnins.2017.00724

**Published:** 2017-12-22

**Authors:** Lindsey B. Shelton, John Koren, Laura J. Blair

**Affiliations:** Department of Molecular Medicine and USF Health Byrd Institute, University of South Florida, Tampa, FL, United States

**Keywords:** Hsp90, aging, tau, chaperones, co-chaperones, proteostasis

## Abstract

The ATP-dependent 90 kDa heat shock protein, Hsp90, is a major regulator of protein triage, from assisting in nascent protein folding to refolding or degrading aberrant proteins. Tau, a microtubule associated protein, aberrantly accumulates in Alzheimer's disease (AD) and other neurodegenerative diseases, deemed tauopathies. Hsp90 binds to and regulates tau fate in coordination with a diverse group of co-chaperones. Imbalances in chaperone levels and activity, as found in the aging brain, can contribute to disease onset and progression. For example, the levels of the Hsp90 co-chaperone, FK506-binding protein 51 kDa (FKBP51), progressively increase with age. *In vitro* and *in vivo* tau models demonstrated that FKBP51 synergizes with Hsp90 to increase neurotoxic tau oligomer production. Inversely, protein phosphatase 5 (PP5), which dephosphorylates tau to restore microtubule-binding function, is repressed with aging and activity is further repressed in AD. Similarly, levels of cyclophilin 40 (CyP40) are reduced in the aged brain and further repressed in AD. Interestingly, CyP40 was shown to breakup tau aggregates *in vitro* and prevent tau-induced neurotoxicity *in vivo*. Moreover, the only known stimulator of Hsp90 ATPase activity, Aha1, increases tau aggregation and toxicity. While the levels of Aha1 are not significantly altered with aging, increased levels have been found in AD brains. Overall, these changes in the Hsp90 heterocomplex could drive tau deposition and neurotoxicity. While the relationship of tau and Hsp90 in coordination with these co-chaperones is still under investigation, it is clear that imbalances in these proteins with aging can contribute to disease onset and progression. This review highlights the current understanding of how the Hsp90 family of molecular chaperones regulates tau or other misfolded proteins in neurodegenerative diseases with a particular emphasis on the impact of aging.

## Introduction

Aging is the biggest risk factor for developing a neurodegenerative disease, but the specific factors which cause these predominantly sporadic diseases are still under investigation (Reeve et al., 2014). As cells within the body age, the cellular homeostasis network must deal with an increasing amount of misfolded and aggregated proteins that can pathogenically accumulate leading to cell death. Aging is caused by compromised cellular homeostasis, fitness, and plasticity, leading to degeneration and cell death in vital organs. According to the “garbage catastrophe” hypothesis, aged differentiated cells lose the capacity to dispose of damaged and malfunctioning proteins (Terman, 2001). Such damaged proteins can assume cytotoxic properties, and their constant removal is thus essential for cell survival. Not only does aging lead to an increased likelihood of protein misfolding and aggregation, it is compounded by a decrease in the efficiency of the protein degradation machinery. The activity of both the proteasome, which is the main mechanism of protein degradation (Rock et al., 1994; Conconi et al., 1996), and chaperone-mediated autophagy (CMA; Cuervo and Dice, 2000b) is significantly impaired with aging and is especially pronounced in post-mitotic cells, such as neurons, potentially resulting in neurodegenerative disease (Terman, 2001). Fortunately, there is a system in place to help the body maintain proteostasis in times of stress and disease: the molecular chaperone network (Söti and Csermely, 2002). This network is comprised of a diverse family of proteins which contains members that are constitutively expressed to help in normal cell maintenance as well as members that become activated during times of stress. All of these chaperones assist in various ways to help fold, refold and degrade misfolded proteins.

The molecular chaperone network is comprised of diverse families of heat shock proteins (Hsps) that are divided based on their molecular mass. The small Hsps regulate general protein aggregation, Hsp40s regulate Hsp70 ATP hydrolysis, Hsp70 folds proteins during translation, and Hsp90 maintains and triages a subset of clients (Liberek et al., 2008; Miyata et al., 2011). While Hsp70 and Hsp90 perform many overlapping roles in the cell, Hsp90 shows more client selectivity. Hsp90 requires ATP to perform these functions including protein degradation, protein folding, prevention of protein aggregation, and protein modification (Echeverría et al., 2011). These regulatory processes are particularly important for intrinsically disordered proteins (IDPs) which have a high propensity to aggregate (Schopf et al., 2017).

Hsp90 binds to one of these IDPs, tau, in a broad region that includes aggregation prone areas (Karagöz et al., 2014). Tau normally functions to stabilize the microtubules and regulate axonal transport (Guo et al., 2017). The pathological accumulation of tau is a hallmark in several neurodegenerative disorders collectively termed tauopathies (Kovacs, 2015); a series of diseases including Alzheimer's disease (AD), progressive supranuclear palsy (PSP), Pick's disease, and chronic traumatic encephalopathy (CTE; Guo et al., 2017). Currently there are no treatment options available which regulate tau pathogenesis (Orr et al., 2017), therefore more work needs to be done to identify potential tau regulating therapeutic strategies.

A promising avenue to target tau is through Hsp90 inhibition. In fact, Hsp90 ATPase-inhibitors rapidly degrade tau aggregates *in vivo* (Dickey et al., 2007a; Luo et al., 2007), but these inhibitors have not yet been successful in clinical trials due to lack of efficacy and associated toxicities (Bhat et al., 2014; Renouf et al., 2016; Thakur et al., 2016). However, Hsp90 regulates tau and other aggregating proteins in coordination with a diverse group of co-chaperones (Schopf et al., 2017). In fact, the levels of many of these co-chaperones have been shown to change with aging, which can alter the fate of tau and potentially contribute to disease onset or severity (Blair et al., 2013; Brehme et al., 2014). It is possible that a more successful treatment strategy may be found by a therapeutic aimed toward regulating these co-chaperones or Hsp90/co-chaperone heterocomplexes (Kamal et al., 2003; Rodina et al., 2016). This review discusses the involvement of Hsp90 and its co-chaperones in disease and how alterations in levels and activity with aging can affect this process (Table 1). Current Hsp90 therapeutic interventions for neurodegenerative diseases will also be reviewed.

**Table 1:** Summary of Hsp90 and Hsp90 co-chaperone levels in aging and Alzheimer's disease (AD).

**Chaperone**	**Gene**	**Function**	**Aging**	**AD**	**References**
Hsp90α	*HSP90AA1*	Chaperone	Repressed	No Data	Brehme et al., 2014
Hsp90ß	*HSP90AB1*	Chaperone	Repressed	Repressed	Brehme et al., 2014
CyP40	*CYP40*	Peptidyl-prolyl isomerase	Repressed	Repressed	Brehme et al., 2014
FKBP51	*FKBP5*	Peptidyl-prolyl isomerase	Induced	Induced	Blair et al., 2013; Brehme et al., 2014
FKBP52	*FKBP4*	Peptidyl-prolyl isomerase	Repressed	Repressed‘	Brehme et al., 2014; Meduri et al., 2016
Xap2	*AIP*	Co-chaperone	Slightly Repressed	No Data	Brehme et al., 2014
PP5	*PPP5*	Ser/Thr phosphatase	Repressed	Activity repressed	Liu et al., 2005; Brehme et al., 2014
FKBP38	*FKBP8*	Peptidyl-prolyl isomerase	Unchanged	No Data	Brehme et al., 2014
FKBP36	*FKBP6*	Peptidyl-prolyl isomerase	Unchanged	No Data	Brehme et al., 2014
WISp39	*FKBPL*	Peptidyl-prolyl isomerase	Unchanged	Repressed	Brehme et al., 2014
Hop	*STIP1*	Client protein maturation	Slightly Repressed	No Data	Brehme et al., 2014
CHIP	*STUB1*	E3 ubiquitin ligase	Unchanged	Unchanged	Brehme et al., 2014
DNAJC7	*DNAJC7*	Steroid receptor co-chaperone	Repressed	Repressed	Brehme et al., 2014
Tom34	*TOMM34*	Mitochondrial import protein	Unchanged	No Data	Brehme et al., 2014
UNC-45A	*UNC45A*	Myosin chaperone	Slightly Induced	Unchanged	Brehme et al., 2014
Tom70	*TOMM70*	Mitochondrial import protein	Repressed	Repressed	Loerch et al., 2008; Brehme et al., 2014
NASP	*NASP*	Co-chaperone	Slightly Induced	Induced	Brehme et al., 2014
SGTA	*SGTA*	Co-chaperone	Unchanged	No Data	Brehme et al., 2014
SGTB	*SGTB*	Co-chaperone	Repressed	Repressed	Loerch et al., 2008; Brehme et al., 2014
Cns1	*TTC4*	Co-chaperone	Induced	No Data	Brehme et al., 2014
CRN	*CRNKL1*	Co-chaperone	Slightly Repressed	No Data	Brehme et al., 2014
Tah1	*RPAP3*	RNA Polymerase II-associated protein	Repressed	No Data	Brehme et al., 2014
TPR1	*TTC1*	Co-chaperone	Unchanged	No Data	Brehme et al., 2014
DYX1C1	*DNAAF4*	Co-chaperone	Induced	No Data	Brehme et al., 2014
AIPL1	*AIPL1*	Co-chaperone	Unchanged	No Data	Brehme et al., 2014
Cdc37	*CDC37*	Inhibits ATPase activity	Unchanged	Repressed	Brehme et al., 2014
Aha1	*AHSA1*	Stimulates ATPase activity	Slightly Repressed	Induced	Brehme et al., 2014; Shelton et al., 2017
p23	*PTGES3*	Inhibits ATPase activity	Slightly Repressed	Unchanged	Brehme et al., 2014
S100A1	*S100A1*	Co-chaperone	No Data	No Data	
FNIP1	*FNIP1*	Co-chaperone	No Data	No Data	

*A summary of the levels of the Hsp90 chaperone network in both aging and AD human samples*.

## Hsp90

Hsp90 is critical to maintaining proteostasis (Brehme et al., 2014) and accounts for up to 6% of all protein within the cell during times of stress (Picard, 2002; Prodromou, 2016). Hsp90 consists of three domains: an N-terminal ATP-binding domain, a middle domain, and a C-terminal domain responsible for the inherent dimerization of the protein (Li and Buchner, 2013). Hsp90 requires ATP in order to dimerize and properly assist in protein folding. The Hsp90 ATPase cycle consists of four stages: the ATP-bound state, an initial intermediate state (I1), a second intermediate state (I2), and finally a closed state in which ATP hydrolysis occurs (Li and Buchner, 2013). There are several different isoforms of Hsp90, however this review will only focus on Hsp90 in the cytosol, which includes Hsp90α (stress-inducible) and Hsp90β (constitutively active) (Li et al., 2012).

The two different cytosolic forms of Hsp90 are 86% genetically identical and have 93% amino acid sequence homology, showing lots of similarities in structure and function. However, there are some differences that set these two isoforms apart. The first difference is the viability of Hsp90 knock-out mice. Mice lacking Hsp90β are embryonically lethal and do not survive past day 9, whereas mice lacking Hsp90α are viable but leads to sterility in male mouse (Table 2; Voss et al., 2000; Grad et al., 2010). There are also some differences in the cellular functions of Hsp90α and Hsp90β. Hsp90α is involved in growth promotion, cell cycle regulation, stress-induced cytoprotection, and cancer cell invasiveness; whereas Hsp90β is involved with early embryonic development, germ cell maturation, cytoskeletal stabilization, cellular transformation, signal transduction, and long-term cell adaptation (Eustace et al., 2004; Sreedhar et al., 2004). While there are some general functional differences between the two cytosolic isoforms more studies are needed to better understand the role of these different isoforms on tau pathology.

**Table 2:** Summary of Hsp90 and Hsp90 co-chaperone knockout mice.

	**Protein**	**Gene**	**KO model**	**Viable**	**Phenotype**	**References**
Hsp90	Hsp90α	Hsp90aa1	Mouse	Yes	Male mice, failure of spermatogenesis; viable and phenotypically normal into adulthood	Grad et al., 2010
	Hsp90β	*Hsp90ab1*	*Mouse*	No	Early embryonic lethality (day E9)	Voss et al., 2000
	Cyp40	*Cyp40*	*Mouse*	Yes	Phenotypically normal	Periyasamy et al., 2010
	FKBP51	*Fkbp5*	*Mouse*	Yes	Resilliant to stress-induced depression-like behavior	Yong et al., 2007; O'Leary et al., 2011; Touma et al., 2011
TPR co-chaperones	FKBP52	*Fkbp4*	*Mouse*	~50% are embryonic lethal	Reduced fertility in both males and females	Cheung-Flynn et al., 2005; Tranguch et al., 2005; Yang et al., 2006
	Xap2	*AIP*	*Mouse*	No	Embryonic lethality	Raitila et al., 2010
	PP5	*Ppp5*	*Mouse*	Yes	Mice survive both embryonic development and into postnatal mice; defect in DNA damage checkpoint after ionizing radiation	Yong et al., 2007
	FKBP38	*Fkbp8*	*Mouse*	No	Embryonically lethal	Bulgakov et al., 2004
	FKBP36	*Fkbp6*	*Mouse*	Yes	Both male and female mice are healthy and live normal lifespans; male mice are sterile	Crackower et al., 2003
	WISp39	*Fkbpl*	*Mouse*	No	Heterozygous FKBPL mice appear normal	Yakkundi et al., 2015
	Hop	*Stip1*	*Mouse*	No	Embryonically lethal around day E9.5-10.5	Beraldo et al., 2013
	CHIP	*Stub1*	*Mouse*	Yes	Develop normally but are susceptible to stress-induced apoptosis of multiple organs; increased peri- and postnatal lethality	Dai et al., 2003
	DnaJC7	*Dnajc7*	*Mouse*	Yes	No information on phenotype	Dickinson et al., 2016
	Tom34	*TOMM34*	*Mouse*	Yes	Phenotypically normal	Terada et al., 2003
	UNC-45A	*UNC45A*	*Mouse*	No	Embryonic lethality	Dickinson et al., 2016
	Tom70	*TOMM70*	*No*	N/A		
	NASP	*NASP*	*Mouse*	No	Embryonic lethality	Richardson et al., 2006
	SGTA	*SGTA*	*Mouse*	Yes	Less fertile with small liters and higher neonatal death rates; smaller body size in both males and females	Philp et al., 2016
	SGTB	*SGTB*	*No*	N/A		
	Cns1	*TTC4*	*Mouse*	Yes	Phenotypically normal	Josefowicz et al., 2012
	CRN	*CRNKL1*	*No*	N/A		
	Tah1	*RPAP3*	*No*	N/A		
	TPR1	*TTC1*	*No*	N/A		
	DYX1C1	*DYX1C1*	*Mouse*	Yes	Embryonic lethality in approx. 2/3; surviving mice develop severe hydrocephalus by postnatal day 16 and died by P21	Tarkar et al., 2013
	AIPL1	*AIPL1*	*Mouse*	Yes	Phenotypically normal	Ramamurthy et al., 2003
Non TPR co-chaperones	Cdc37	*Cdc37*	*C. elegan*	No	Embryonically lethal in C. elegans	Beers and Kemphues, 2006
	Aha1	*Ahsa1*	*Mouse*	Yes	No information on phenotype	The Jackson Laboratory: Stock No: 029805
	p23	*Ptges3*	*Mouse*	No	Perinatal lethality resulting from defective lung development; Abnormal skin and reduced expression of GR markers	Grad et al., 2006; Lovgren et al., 2007; Nakatani et al., 2007
	S100A1	*S100A1*	*Mouse*	Yes	Phenotypically normal	Du et al., 2002
	FNIP1	*FNIP1*	*Mouse*	Yes	Phenotypically normal	Hasumi et al., 2015

*This table contains information on the available knock-out mouse lines for the Hsp90 chaperone family*.

Alterations in chaperone expression are commonly seen in aging, leading to complications within the Hsp90 chaperone network. In fact, recent work has shown the levels of many Hsp90 co-chaperones are also altered in the aged brain (Brehme et al., 2014). These co-chaperones are necessary for client selection and triage. There are two main categories of Hsp90 co-chaperones: tetratricopeptide repeat (TPR) and non-TPR containing (Prodromou et al., 2000). Therefore, changes in Hsp90 levels are part of a larger imbalance in the chaperone network which contribute to aging and age-related neurodegenerative disorders.

## TPR co-chaperones

**Figure 1:**
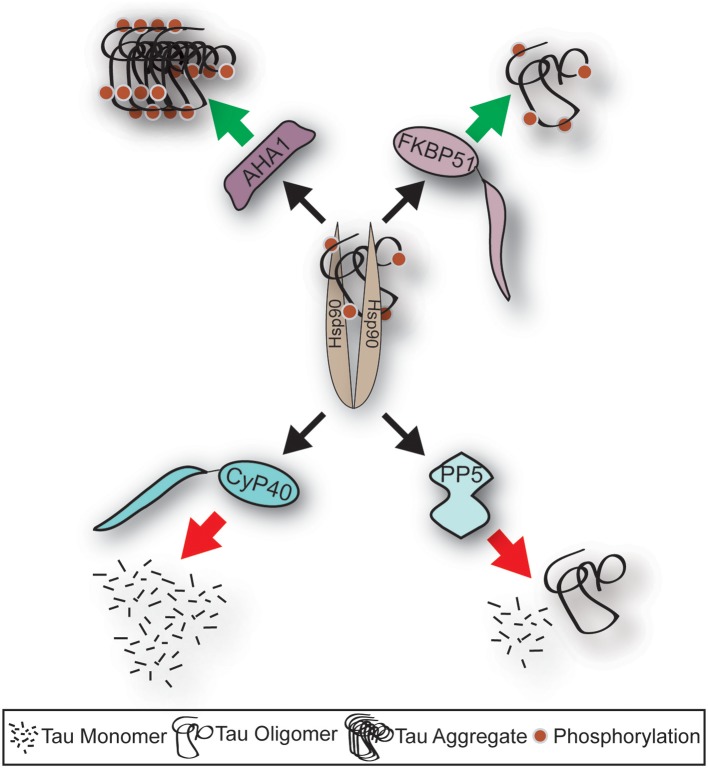
Schematic depicting fate of tau following Hsp90 interaction with distinct co-chaperone; the impact of Alzheimer's disease on the levels of co-chaperones. Aha1 and FKBP51 protein levels are induced in AD, and their association to tau leads to increased aggregation. Whereas, CyP40 and PP5 levels are repressed in AD, and their association to tau leads to reduced tau aggregation. This schematic highlights the important role of co-chaperones in AD.

TPR-containing Hsp90 co-chaperones interact with the C-terminal MEEVD peptide motif on Hsp90 (Li et al., 2012). Since Hsp90 functions as a dimer, two TPR-containing co-chaperones could interact simultaneously. However, these interactions are dependent on the isoform of Hsp90 and the repertoire of expressed co-chaperones. In fact, co-chaperones do compete for binding to Hsp90 (Harst et al., 2005; Hildenbrand et al., 2011). This competition can have beneficial or detrimental effects on tau pathology. Known examples include co-chaperones which interact with Hsp90 to promote the degradation of aberrant tau or others which drive tau oligomerization and aggregation. Therefore, an imbalance in protein levels with aging and AD compound this already complex competition for binding Hsp90 to regulate tau fate (Figure 1). Here, we will describe these TPR-co-chaperones, and how their interaction with Hsp90 regulates tau.

### Immunophilins and immunophilin homologs

Hsp90 interacts with six immunophilins that display peptidyl-prolyl isomerase (PPIase) activity through TPR domains including cyclophilin 40 (CyP40) and five FK506-binding proteins: FKBP51, FKBP52, FKBP36, FKBP38, FKBPL/WISp39 (Jascur et al., 2005; Jarczowski et al., 2009; Guy et al., 2015; Blundell et al., 2017). These PPIases regulate the twisting of proline bonds through stabilization of the *cis*-*trans* transition state and accelerate the isomerization process. This is particularly important for tau, which has 40 proline residues that regulate phosphorylation and aggregation propensity (Mandelkow and Mandelkow, 2012). Hsp90 also interacts with two immunophilin homologs: protein phosphatase 5 (PP5) and XAP2/FKBP37. Altered levels of many of these immunophilins and immunophilin-like proteins have been found in aging and AD (Table 1), which could skew the competition dynamics for Hsp90 binding (discussed later in this review) and may promote toxic tau accumulation.

#### CyP40

An interesting PPIase, CyP40, decreases in aging and is further repressed in AD (Table 1; Brehme et al., 2014). CyP40 was recently shown to disaggregate tau fibrils *in vitro* and prevents toxic tau accumulation *in vivo* preserving memory, demonstrating a neuroprotective role for CyP40 in the brain (Baker et al., 2017). The PPIase activity of CyP40 is slightly repressed when bound to Hsp90, but under cellular stress CyP40 can release from Hsp90 increasing its isomerase and chaperone activity (Blackburn et al., 2015). However, as CyP40 levels decrease with aging, it is possible that the pool of free CyP40 is not sufficient to help disentangle aggregating proteins, like tau.

#### FKBP51

Contrary to the neuroprotective effects of CyP40, two FK506-binding proteins (FKBPs) have been shown to stimulate toxic tau aggregation (Blair et al., 2013; Giustiniani et al., 2015; Kamah et al., 2016). One of these, FKBP51, coordinates with Hsp90 to preserve toxic tau oligomers *in vivo* (Blair et al., 2013). In fact, mice lacking FKBP51 have decreased tau levels in the brain (Jinwal et al., 2010; Blair et al., 2013). However, throughout aging, FKBP51 levels progressively increase and are further increased in AD brain samples (Table 1; Blair et al., 2013; Sabbagh et al., 2014). Previous studies have also shown that FKBP51 can form complexes with tau in both human AD brain samples and control samples (Jinwal et al., 2010). Additionally, this study showed that FKBP51 was able to stabilize microtubules, suggesting a novel and unique function for FKBP51 (Jinwal et al., 2010). Taken together, the increase in FKBP51 in aging and AD suggest that targeting FKBP51 could offer a potential therapeutic strategy for tauopathies such as AD.

#### FKBP52

FKBP52 interacts both physically and functionally with tau and promotes tau aggregation *in vitro* (Giustiniani et al., 2015; Meduri et al., 2016). FKBP52 induces oligomers from both P301L and truncated wild-type tau. Interestingly, this oligomerization is not due to the PPIase activity of FKBP52, instead the oligomerization of tau appears to occur via molecular interaction (Kamah et al., 2016). FKBP52 can also induce aggregation of a truncated form of tau that appears to have prion like behavior, suggesting a possible mechanism for the spread of tau pathology throughout the brain in diseases such as AD (Giustiniani et al., 2015). However, it is interesting to note that FKBP52 levels are lower in the cortex of AD patients' brains (Table 1; Brehme et al., 2014; Meduri et al., 2016).

#### FKBP36, FKBP38, and FKBPL

There are several other FKBPs that act as co-chaperones to Hsp90 including FKBP36, FKBP38, and FKBPL (WISp39), however their relationship to tau, if any, is still unknown at this point and so they will not be discussed in detail.

#### XAP2

XAP2, otherwise known as FKBP37 or Aryl hydrocarbon receptor interacting protein (AIP), contains a PPIase homologous domain. While a direct role of XAP2 in tau pathogenesis has not been described, studies have shown that XAP2 is activated by histone deacetylase (HDAC) 6, which has been linked to pathogenic tau (Kekatpure et al., 2009; Cook et al., 2012; Selenica et al., 2014). In addition, XAP2 coordinates with Hsp90 to regulate glucocorticoid receptor signaling (Laenger et al., 2009), which has also been implicated in the production of pathogenic tau (Pinheiro et al., 2016). Additional studies are needed to determine the direct or indirect effects of XAP2 on tau pathology.

#### PP5

Another member of this family, protein phosphatase 5 (PP5), is repressed in aging. PP5 is contains a PPIase homology domain, but does not display classical PPIase activity, since it can bind FK506 (Silverstein et al., 1997). Instead, PP5 acts as a Ser/Thr phosphatase, which activates when bound to Hsp90 (Conde et al., 2005). PP5 activity has been shown to be repressed in AD (Table 1; Liu et al., 2005). Studies have shown that PP5 is able to dephosphorylate tau at several phosphorylation sites connected to AD pathology (Gong et al., 2004). Further studies are needed to better understand if the upregulation of PP5 could be used to slow or prevent tau pathogenesis.

#### Hop

The Hsp70-Hsp90 organizing protein, otherwise known as Hop and sometimes STIP1 (stress inducible protein 1), is involved in helping transfer client proteins from the early stages of protein maturation involving Hsp70 and Hsp40 to the later stages of the cycle involving Hsp90 (Baindur-Hudson et al., 2015). As such, Hop plays a crucial role in the maturation of client proteins, like tau. A previous study found that when Hop was depleted using siRNA, there was an accumulation of tau (Jinwal et al., 2013). This suggests that Hop is necessary for tau clearance via Hsp70/Hsp90. In fact, loss-of-function mutations in Hop drive toxic tau accumulation in a fly model of tauopathy (Ambegaokar and Jackson, 2011). Together these studies demonstrate a protective role of Hop in a tauopathic brain.

#### CHIP

C-terminus of Hsc70-interacting protein (CHIP) is highly involved in the Hsp70-Hsp90 machinery acting not only as a co-chaperone, but also as an E3 ubiquitin ligase responsible for ubiquitin-dependent proteasomal degradation (Edkins, 2015). CHIP has many roles within the cell including stress-response activation, protein triage, and restitution of the stress response (Dickey et al., 2007b). CHIP has been linked to several neurodegenerative disorders including Huntington's disease, Parkinson's disease and AD as well as other diseases such as cystic fibrosis and cancer (Dickey et al., 2007b; Edkins, 2015). In tauopathic mice, CHIP regulates the removal of tau species that have undergone abnormal phosphorylation and folding (Dickey et al., 2007b). Additionally, silencing CHIP via siRNA, led to a massive increase in tau levels (Dickey et al., 2008). Similarly, CHIP/*Stub1*-knockout mice have increased accumulation of phospho- and total tau species (Table 2; Palubinsky et al., 2015). Overexpression of CHIP could represent a therapeutic strategy to prevent neuronal cell death and improve outcomes of neurodegenerative diseases by promoting the degradation of tau.

#### DnaJC7

DnaJC7, also known as Tpr2, simultaneously binds Hsp70 and Hsp90 via its two TPR domains (Brychzy et al., 2003). To date, a link between DnaJC7 and tau has not been investigated. However, it is known that DnaJC7 plays an important role in steroid receptor chaperoning, as well as recycling substrates from Hsp90 back to Hsp70 via this unique TPR interaction (Brychzy et al., 2003; Moffatt et al., 2008). Additional studies are needed to understand if DnaJC7 regulates tau pathogenesis.

#### Tom34

Tom34 is a co-chaperone involved in mitochondrial protein import. One study found that in *Drosophila*, impaired Tom34 gene function led to enhanced tau pathology (Ambegaokar and Jackson, 2011). Conversely, the same study demonstrated Tom34 overexpression was able to suppress tau toxicity elucidating a role for Tom34 in tau pathology in *Drosophila*. The mechanism by which Tom34 promotes tau pathology remains unclear. It is possible that mitochondrial dysfunction could lead to cellular stress which, in turn, could enhance tau pathology. Additional studies are needed to fully elucidate this interaction.

In addition, there are other TPR-containing Hsp90 co-chaperones such as UNC-45, Tom70, NASP, SGTA, SGTB, Cns1, CRN, Tah1, TPR1, DYX1C1, and AIPL1. However, very little is known about most of these co-chaperones in the brain and even less is known about their interactions with tau, therefore they will not be discussed in detail in this review.

### Non-TPR co-chaperones

#### Cdc37

Cell division cycle 37 (Cdc37) slows the ATPase activity of Hsp90 allowing a prolonged interaction between Hsp90 and its client proteins (Cox and Johnson, 2011). Cdc37 is also required for the stable folding of protein kinases in coordination with Hsp90 (Calderwood, 2015). Many of these kinases are known to phosphorylate tau at sites associated with AD, such as GSK3β and MAPK13 (Taipale et al., 2012; Jin et al., 2016). Interestingly, overexpression of Cdc37 preserves tau, and its suppression reduces tau (Jinwal et al., 2012). However, additional studies are needed to better understand the dynamics between Cdc37 and tau phosphorylation.

#### Aha1

The activator of Hsp90 ATPase homolog 1 (Aha1) works as a co-chaperone to stimulate the ATPase function of Hsp90 to regulate the folding and activation of client proteins. Aha1 interacts with Hsp90 independent of its nucleotide status and allows the Hsp90 ATPase cycle to skip the I1 phase, thus accelerating the progression of the ATPase cycle dramatically (Li and Buchner, 2013; Wolmarans et al., 2016). Aha1 levels have been shown to increase with AD. In fact, we have found that Aha1 levels in the medial temporal gyrus of human brain correlated with increased tau Braak staging (Shelton et al., 2017). In the same study, we found that high levels of Aha1 in a tau transgenic mouse model increased tau oligomers as well as neuronal loss concomitant with cognitive deficits (Shelton et al., 2017). Since Aha1 levels are repressed in aging, but are abnormally preserved in AD, tau aggregation could be accelerated in part by Aha1 in the AD brain. Previous studies have also implicated Aha1 for a role in cystic fibrosis. In fact, one study showed that knockdown of Aha1 promotes the translocation of the disease-related mutant of cystic fibrosis transmembrane conductance receptor, CFTR, to the plasma membrane, allowing it to properly function (Wang et al., 2006). Thus, treatments which reduce Aha1 may be beneficial for both AD and cystic fibrosis.

#### P23

p23 has an opposing effect on Hsp90 compared to Aha1. p23 works by inhibiting the ATPase activity of Hsp90. The interaction between Hsp90 and p23 is nucleotide-dependent meaning that p23 can only interact with Hsp90 when ATP is bound (Sullivan et al., 1997). p23 works in a unique way to inhibit ATPase activity, it can either inhibit the hydrolysis process or it can impede the release of ADP and Pi (Rehn and Buchner, 2015). As a co-chaperone, p23 works to suppress protein aggregation and exhibits chaperoning activity, although p23 is not able to refold proteins on its own (Freeman et al., 1996). Inhibition of p23 in an siRNA screen of Hsp90 co-chaperones showed that silencing p23 reduced both total and phospho-tau (Jinwal et al., 2012, 2013). p23 also plays an important role in preventing endoplasmic reticulum (ER) stress-induced cell death, which can be triggered by misfolded proteins, like tau (Rao et al., 2006; Abisambra et al., 2013). However, p23 can be cleaved during ER stress-induced cell death into a smaller p19 fragment which is then unable to exert its anti-apoptotic effects (Zhang et al., 2013). A mutant p23 (p23D142N) that is uncleavable was shown to ameliorate the ER stress-induced cell death *in vitro* and suggests that this mutant p23 could be a potential therapeutic target in neurodegenerative diseases (Zhang et al., 2013).

#### S100A1

S100 calcium-binding protein A1 (S100A1) interacts with Hsp90. One study used siRNA to screen several Hsp90 co-chaperones to investigate the effect on tau. This study found that reductions in S100A1 also led to massive reductions in both phospho- and total tau levels in cells (Jinwal et al., 2013). S100A1 could play a role in stabilizing tau, thus leading to a worsening of tau pathology. Therefore, silencing or knocking down S100A1 could offer a potential therapeutic strategy for tauopathies.

#### FNIP1

The folliculin-interacting protein 1 (FNIP1) is able to interact with Hsp90 as a co-chaperone in order to inhibit its ATPase activity. One study found that FNIP1, in complex with FNIP2 and Hsp90, was able to stabilize the tumor suppressor folliculin (FLCN; Woodford et al., 2016). FNIP1 was shown to interact directly with Hsp90, and it can also interact with other co-chaperones such as p23, Hop and Cdc37 (Woodford et al., 2016). FNIP1 was also shown to compete for binding with Aha1 suggesting an important role for FNIP1 in the Hsp90 chaperone network (Woodford et al., 2016). Additional studies are needed to determine if FNIP1 could regulate tau directly or potentially through competition with Aha1 to bind Hsp90 and alter its ATPase activity.

### Aging in the Hsp90 chaperone network

All of the above mentioned co-chaperones interact with Hsp90 in order to form diverse heterocomplexes, however, changes in Hsp90 expression with aging can alter their composition. There is conflicting data on Hsp90 levels with age in both human and animal studies. One study focused on the basal levels of cytosolic Hsp90 in peripheral blood mononuclear cells (PBMC) and found that in aged human samples there was an increase in Hsp90 under normal physiological conditions when compared to young samples (Njemini et al., 2007). Another study had similar findings in the hippocampus of aged gerbils. This study demonstrated that cytosolic Hsp90 levels were significantly increased in the hippocampus of aged gerbils (24 months) compared to adult gerbils (6 months) (Lee et al., 2011). Conversely, there are also studies showing decreased levels of Hsp90 in aged human brain samples. For instance, two other studies investigated the levels of chaperone proteins in the human brain. One study found that cytosolic Hsp90 was repressed in the superior frontal gyrus, while another demonstrated a similar repression in the prefrontal cortex of aged patients compared to controls (Berchtold et al., 2008; Loerch et al., 2008; Brehme et al., 2014). Taken together, this data suggests that alterations in Hsp90 levels do not occur uniformly and that changes in the expression of Hsp90 with aging may vary between cell types and brain regions (Berchtold et al., 2008). While Hsp90 protein levels are an important factor with aging, co-chaperone expression levels could be equally important in heterocomplex formation.

In addition to the differences in expression levels of Hsp90, there are also changes in expression levels of co-chaperone proteins during the aging process. Almost all of the Hsp90 co-chaperones are repressed in aging, suggesting that these proteins could play important roles in maintaining homeostasis within the cell (Brehme et al., 2014). For instance, CyP40, FKBP52, PP5, Hop, p23, and Aha1 are all repressed in the aged brain. All of these proteins are integral to the Hsp90 chaperone system and when levels of these proteins go down the Hsp90 chaperone network can no longer function normally, which can lead to an increased risk of developing a neurodegenerative disease. Interestingly, one co-chaperone is significantly induced in the aged brain and that is FKBP51. FKBP51 has several important roles within the cell including immunoregulation as well as helping with protein folding and trafficking in complex with Hsp90. Because FKBP51 is induced in aging, while many other co-chaperones are reduced, this suggests that the imbalance seen in these proteins during aging could lead to completely different Hsp90 heterocomplexes resulting in the dysfunction of cellular homeostasis during aging.

Hsp90 is able to form many unique heterocomplexes with different co-chaperones in order to regulate protein triage. Hsp90 heterocomplexes are unique in that there is usually a specific progression of co-chaperones that interact with Hsp90 (Schopf et al., 2017). One interesting aspect to these heterocomplexes is the fact that Hsp90 can bind multiple co-chaperones simultaneously. One study found that Hsp90 could form stable complexes with Hsp90, FKBP52, Hop, and p23 (Hildenbrand et al., 2011). There does appear to be a hierarchy though, with some co-chaperones able to bind more strongly than others. For example, Aha1 has been shown to compete with Hop, FNIP1, and p23 for the ability to bind with Hsp90 (Harst et al., 2005; Woodford et al., 2016). These competition dynamics between Aha1 and p23/FNIP1 suggest that there is a constant battle for control of the ATPase activity of Hsp90. Additionally, FKBP51 and FKBP52 have been shown to have greater relative binding to Hsp90 compared to other TPR co-chaperones (Schülke et al., 2010). While not as strong as FKBP51 and FKBP52, PP5 forms more complexes with Hsp90 than most other TPR co-chaperones. Taken together, increased FKBP51 and decreased PP5 and CyP40 could contribute to an imbalance in Hsp90 heterocomplexes which may promote increased tau phosphorylation and aggregation causing neurotoxicity (Blair et al., 2013). This suggests an even more complex system in place because depending on the amount of certain co-chaperones and their relative ability to bind to Hsp90; certain maladaptive complexes could be more abundant than others with aging.

In addition to altering Hsp90 heterocomplex composition and client selection, altered Hsp90 co-chaperone expression can interfere with degradation of aberrant proteins via the proteasome or autophagy. As mentioned above, aged cells are often inundated with misfolded and aggregated proteins, which can overload the Hsp90 chaperone network causing a negative spiral where there are not enough healthy chaperone molecules to refold or degrade aberrant proteins. In addition to the problems faced with an overwhelmed chaperone network, the proteolytic activity of the proteasome also declines with aging, and in fact Hsp90 has been shown to protect the proteasome from age-related, oxidative-dependent decline (Conconi and Friguet, 1997). However, with advanced aging, the association between Hsp90 and the proteasome drastically decreases (Conconi et al., 1996). This suggests that because the Hsp90 chaperone system and the proteasome are so connected, when one starts to fail the other will fail as well leading to cytotoxicity and cell death. Proteins can also be degraded by CMA; however, CMA activity also decreases with age (Cuervo and Dice, 2000a). Hsp90 and Hop are both involved in the CMA system; helping to unfold the target substrate before it can translocate into the lysosome for degradation. As mentioned previously, both Hsp90 and Hop are repressed in aging and therefore may not be able to assist in the translocation of substrates, leading to a buildup of misfolded or aggregated proteins. Post-translational modifications (PTMs) of Hsp90 can also complicate the matter further.

There are many different PTMs that can affect Hsp90 including phosphorylation, acetylation, S-nitrosylation, oxidation, and ubiquitination; and all of these PTMs can impact the chaperoning function of Hsp90. Phosphorylation of Hsp90 leads to reduced chaperoning ability and phosphorylation of specific tyrosine residues can affect the ability of Hsp90 to interact with distinct client proteins (Zhao et al., 2001; Mollapour and Neckers, 2012). Acetylation of Hsp90 affects client protein interaction and also decreases binding of Hsp90 to ATP (Yu et al., 2002; Mollapour and Neckers, 2012). S-nitrosylation, oxidation and ubiquitination also inhibit Hsp90 chaperone activity (Blank et al., 2003; Martínez-Ruiz et al., 2005; Chen et al., 2008). These PTMs increase with aging and can alter the ability of Hsp90 to function properly as well as change the ability of different co-chaperones to bind. As the chaperone network declines with aging, so does the ability of the cell to recover from damaged proteins and stress, thus leading to an environment which promotes aberrant protein accumulation and neurotoxicity.

### Targeting the Hsp90 chaperone network

Inhibition of the ATPase activity of Hsp90 has been shown to have positive outcomes in cell culture and animal models of tauopathy. Hsp90 ATPase inhibitors have been developed to target each of the three domains; with the majority of Hsp90 inhibitors targeting the N-terminal domain (Bhat et al., 2014). Inhibition of Hsp90 induces the expression of protective chaperones, Hsp70 and Hsp40, further promoting the degradation of aberrant proteins (Carman et al., 2013). Previous studies have shown that Hsp90 inhibition decreased the levels of hyperphosphorylated and/or mutated tau species both in cells and mice. The Hsp90 N-terminal domain inhibitor, EC102, was used to demonstrate degradation of hyperphosphorylated pathologically relevant tau in cells (Dickey et al., 2007a). Another N-terminal Hsp90 ATPase inhibitor, 17-AAG, was shown to decrease levels of phosphorylated tau in cells, and a related N-terminal Hsp90 ATPase inhibitor, PU-DZ8, reduced soluble and insoluble tau in tauP301L mice (Luo et al., 2007). Although Hsp90 inhibitors have been in clinical development since 1999, none have reached New Drug Application (NDA) status (Bhat et al., 2014). All of these clinical trials were focused on investigating Hsp90 inhibitors on various cancers. Hsp90 plays a similar role in both neurodegenerative disorders and cancer, however because of the complexity of the brain and the need for a blood-brain barrier (BBB) permeable drug, the clinical development of Hsp90 inhibitors for neurodegenerative diseases has been even less successful. While the development of Hsp90 inhibitors is still underway, it is possible that the development of therapeutics which target Hsp90 heterocomplexes or discrete Hsp90 co-chaperones could open up additional avenues for success in developing a BBB-permeable drug.

In addition to offering more potential therapeutic targets, small molecules which modify Hsp90/co-chaperone interactions may also show more specificity for a specific pool of Hsp90 which may reduce the number of on-target side effects. There are very few Hsp90 co-chaperone targeting small molecules, and of these, only a handful of these have been investigated for their role in effecting tau. There is a Hop/Hsp90 complex specific inhibitor, C9, however, there is no available data on how chemical inhibition of this complex affects tau accumulation (Pimienta et al., 2011). The Cdc37/Hsp90 inhibitors, Celasterol and Withaferin A (Zhang et al., 2008; Yu et al., 2010), reduce tau levels and a new compound, platycodin D has just been discovered (Li et al., 2017). Platycodin D does not affect the ATPase activity of Hsp90, but instead disrupts the interaction between Hsp90 and Cdc37 leading to client protein degradation without an increase in Hsp70 (Li et al., 2017). More work still needs to be done to better understand the role of Cdc37 in tau phosphorylation to determine if targeting this complex is of therapeutic benefit. Developing drugs to target discrete FKBPs has been challenging due to their homology, however, despite their structural similarity they do display differences in conformational flexibility which could be a way to potentially target specific FKBPs in the future (LeMaster and Hernandez, 2015). Interestingly, one study demonstrated that patients chronically treated with FK506, which inhibits the PPIase domain of many of the FKBPs, significantly reduced the incidence of AD (Taglialatela et al., 2015). The targeting FKBP51 is of great interest for the treatment of tauopathies as well as mood disorders (Zannas and Binder, 2014). Recently a PPIase antagonist has been developed which shows selectivity for FKBP51, but additional studies are needed to determine if targeting the PPIase domain of FKBP51 will be effective in regulating tau accumulation (Jinwal et al., 2010). There is one compound, MJC13, which targets the FKBP52-Hsp90-androgen receptor complex (De Leon et al., 2011). MJC13 results in a reduced stress response which has demonstrated therapeutic potential in cancer, but so far MJC13 has not been investigated for a role in tau pathology (De Leon et al., 2011). Additionally, Aha1-specific inhibitors have been recently developed (Hall et al., 2014). One of these inhibitors, KU-177, reduced insoluble tauP301L levels in cells (Shelton et al., 2017). While this is an exciting result, more studies are needed to determine if Aha1 inhibitors regulate tau similarly *in vivo*. There is still much to be done to develop compounds which target the Hsp90 chaperone network, but there are a lot of promising leads which can be targeted to develop disease-modifying therapeutics.

## Conclusions

The Hsp90 chaperone machinery plays a huge role in both aging and neurodegenerative diseases. Hsp90 is one of the most highly expressed proteins in the cell and is involved in a myriad of cellular processes. Previous work has focused on inhibition of Hsp90 to triage misfolded proteins. There are also many co-chaperones that associate with Hsp90 and play their own roles in aging and neurodegeneration. As these Hsp90 co-chaperones change with age they can significantly impact the propensity for certain neurodegenerative diseases. FKBP51 steadily increases with age and both FKBP51 and Aha1 are induced in the AD brain suggesting that these two co-chaperones negatively affect tau pathology. On the other hand, both CyP40 and PP5 are repressed in aged and AD brains. CyP40 disaggregates tau fibrils *in vitro* and PP5 dephosphorylates tau restoring microtubule binding, suggesting that increasing the levels or activity of these co-chaperones could have a beneficial, neuroprotective role in diseases such as AD. These are just a few examples of how important maintaining the balance of Hsp90 co-chaperones is to homeostasis, and what can happen when they are altered in aging and disease. Hsp90 co-chaperones offer a unique target for potential therapeutics due to their specific roles within the Hsp90 machinery. Overall, more work needs to be done to develop BBB-permeable therapeutics to target discrete Hsp90 co-chaperones for the treatment of AD and other tauopathies.

## Author contributions

LS wrote the manuscript. JK created the figures and edited the manuscript. LB critiqued and edited the manuscript drafts.

### Conflict of interest statement

The authors declare that the research was conducted in the absence of any commercial or financial relationships that could be construed as a potential conflict of interest.
